# HIF‐1α/Netrin‐4 Axis Mediates RIPC‐Induced Angiogenesis and Neurogenesis After Ischemic Stroke

**DOI:** 10.1111/jcmm.71121

**Published:** 2026-04-05

**Authors:** Zhaowei Feng, Zhenqian Liu, Siyu Tang, Meihua Pan, Kaishen Zhu, Yiwei Liu, Chunyu Wang, Ruiqin Yao, Xiue Wei, Haiyan Liu

**Affiliations:** ^1^ Department of Neurology The Second Affiliated Hospital of Xuzhou Medical University Xuzhou Jiangsu Province China; ^2^ Xuzhou Key Laboratory of Neurobiology, Department of Cell Biology and Neurobiology Xuzhou Medical University Xuzhou Jiangsu Province China; ^3^ Biology, College of Arts and Sciences The Ohio State University Columbus Ohio USA

**Keywords:** angiogenesis, cerebral ischemia/reperfusion, HIF‐1α, neurogenesis, NTN4, remote ischemic postconditioning

## Abstract

Remote ischemic postconditioning (RIPC) confers neuroprotection in ischemic stroke partly via promoting angiogenesis and neurogenesis, but its precise molecular mechanisms remain unclear; here, we investigated the role of the secreted guidance protein Netrin‐4 (NTN4) and its upstream regulator hypoxia‐inducible factor 1α (HIF‐1α) in mediating RIPC's reparative effects, using endothelial‐specific Ntn4 knockout (KO) mice subjected to transient middle cerebral artery occlusion (MCAO) and RIPC, alongside in vitro assays with brain microvascular endothelial cells (BMECs) and neural stem cells (NSCs) and molecular interaction analyses, including DNA pull‐down and chromatin immunoprecipitation (ChIP), finding that RIPC significantly upregulated NTN4 expression in the ischemic penumbra of MCAO mice, that endothelial‐specific Ntn4 knockout abolished RIPC's protective effects—impairing neurological recovery, angiogenesis and neurogenesis, which were rescued by recombinant NTN4 administration, that NTN4 promoted BMEC proliferation and tube formation via an integrin β1‐PI3K/AKT pathway while conditioned medium from Ntn4‐overexpressing BMECs enhanced NSC neuronal differentiation through an integrin β1‐MAPK/ERK axis, and that RIPC stabilised HIF‐1α, which directly bound the Ntn4 promoter to drive its transcription, collectively establishing that RIPC orchestrates brain repair by stabilising HIF‐1α to transcriptionally activate endothelial NTN4, which signals through integrin β1 to drive parallel PI3K/AKT and MAPK/ERK pathways for angiogenesis and neurogenesis, highlighting this axis as a key therapeutic target in stroke.

## 1 Introduction

Ischemic stroke remains a leading cause of central nervous system (CNS) dysfunction, characterised by high morbidity, mortality and long‐term disability [1]. Notably, its incidence among young adults (≤ 55 years) has risen sharply in recent years [2]. While pharmacological and mechanical thrombolysis have significantly improved acute stroke outcomes, survivors frequently experience persistent neurological deficits, including epilepsy, chronic pain, cognitive impairment and depression [3, 4]. Developing neuroprotective strategies to enhance functional recovery and elucidating their molecular mechanisms remain critical challenges.

Emerging evidence indicates that remote ischemic postconditioning (RIPC) effectively promotes poststroke rehabilitation [5]. This intervention mitigates cerebral injury and enhances neurobehavioral recovery by reducing infarct volume, preserving blood–brain barrier (BBB) integrity and stimulating neuronal survival, neurogenesis and angiogenesis [6, 7, 8, 9]. The neuroprotective effects of RIPC involve multiple mechanisms, such as suppressing neuroinflammation, modulating glial activation, balancing immune cell populations and regulating key signalling pathways [10]. Specifically, RIPC downregulates astrocytic aquaporin‐4 (AQP4) [11], reduces neuronal hypoxia‐inducible factor‐1α (HIF‐1α) expression [9], activates peroxisome proliferator‐activated receptor γ (PPARγ), inhibits STAT3 overactivation [12] and elevates tissue kallikrein (TK) levels [13]. Despite its demonstrated benefits for neurogenesis and angiogenesis [6, 8], the precise mechanisms underlying RIPC's effects require further investigation.

Netrin‐4 (NTN4), a member of the Netrin family, functions as an axon guidance molecule across vertebrates and invertebrates [14]. Recent studies highlight its role in developmental and therapeutic angiogenesis, where it enhances endothelial cell migration, proliferation and tube formation in hindlimb ischemia models [15]. Intracerebroventricular NTN4 administration improves functional recovery after middle cerebral artery occlusion (MCAO) by stimulating vascular proliferation [16]. Paradoxically, NTN4 exhibits antiangiogenic activity in pathological angiogenesis models like laser‐induced choroidal neovascularisation [17]. It also counteracts endothelial senescence and inflammation [18]. Notably, NTN4 levels rise significantly in ischemic rat brains from 24 h to 7 days post‐injury [16], yet the regulatory mechanisms controlling its expression remain unknown.

This study examined whether RIPC's neuroprotection in ischemic stroke involves NTN4 upregulation. Using an MCAO mouse model, we first assessed NTN4 expression changes following RIPC. We then employed Ntn4 transgenic mice (Tie2‐cre: *Ntn4* ^f/f^) to evaluate NTN4's role in neurological recovery, neurogenesis and angiogenesis, testing whether RIPC's effects depend on NTN4. Given HIF‐1α's established regulation of Vascular Endothelial Growth Factor (VEGF) and Netrin‐1 in angiogenesis and neurogenesis [19, 20, 21], we further demonstrated that RIPC elevates NTN4 expression via HIF‐1α signalling. These findings identify NTN4 as a novel mediator of RIPC's therapeutic effects and a potential target for ischemic stroke treatment.

## 2 Methods

### 2.1 Animals

Specific‐pathogen‐free (SPF) adult male C57BL/6J mice (8–10 weeks old, 25–30 g) were obtained from the Experimental Animal Center of Xuzhou Medical University and used to maintain a wild‐type colony. The day of birth was designated as postnatal day 0 (P0). *Ntn4*‐floxed (*Ntn4* ^f/f^; GemPharmatech, Strain No. T015342), Tie2‐Cre (Strain No. T003764) and Tie2‐CreERT2 (Strain No. T004737) mice were purchased from GemPharmatech (Nanjing, China). To generate endothelial cell (EC)‐specific *Ntn4* conditional knockout (cKO) mice, Tie2‐Cre or Tie2‐CreERT2 mice were crossed with *Ntn4* ^f/f^ mice. The breeding strategy involved crossing Tie2‐Cre; *Ntn4* ^f/f^ or Tie2‐CreERT2; *Ntn*4^f/f^ mice with *Ntn4* ^f/f^ mice to produce experimental (Tie2‐Cre; *Ntn4* ^f/f^ or Tie2‐CreERT2; *Ntn*4f^/f^) and control (*Ntn4* ^f/f^) littermates. Genotyping was performed by PCR analysis of genomic DNA extracted from tail biopsies [22]. All mice were housed under SPF conditions with a 12/12‐h light/dark cycle and provided with food and water ad libitum. All animal experiments were approved by and conducted in accordance with the guidelines of the Institutional Animal Care and Use Committee of Xuzhou Medical University (Approval No. 202101A018), following National Institutes of Health standards and WMA Declaration of Helsinki principles. This study did not involve human participants.

### 2.2 Middle Cerebral Artery Occlusion Model Establishment

Focal cerebral ischemia was induced by transient intraluminal MCAO in adult male C57BL/6J mice (8–10 weeks old). Mice were anaesthetised with 1.5% isoflurane and placed in a supine position. Following a midline neck incision, the left common carotid artery (CCA), external carotid artery (ECA) and internal carotid artery (ICA) were carefully exposed and isolated. The proximal ends of the CCA and ECA were temporarily clamped. A small incision was made in the CCA, and a silicone‐coated monofilament (diameter: 0.18 mm) was inserted and gently advanced into the ICA until mild resistance was felt, indicating occlusion of the middle cerebral artery. After 2 h of occlusion, the filament was withdrawn to allow reperfusion. Finally, the CCA was permanently ligated, and the surgical wound was sutured.

### 2.3 Cannula Infusion Experiment

Mice were anaesthetised with isoflurane using a precision vaporiser and positioned in a stereotaxic frame. A guide cannula (RWD) was stereotaxically implanted into the left lateral ventricle (coordinates: AP = −0.3 mm, ML = +1.0 mm, DV = −2.3 mm) and secured to the skull with dental cement. A dummy cannula (obturator) was inserted into the guide cannula post‐surgery to maintain patency. For drug administration, recombinant mouse NTN4 protein (Yeasen, 93250ES60) was dissolved in sterile 0.9% saline (2 μg/μL). Prior to infusion, the dummy cannula was removed and the guide cannula was checked for patency using a pre‐sharpened injector. A 1 μL volume of the drug solution was then infused into the lateral ventricle at a rate of 0.1 μL per 2.5 min via a microsyringe (Baige) connected to a sharpened injector cannula. Following infusion, the injector was left in place for an additional 5 min to minimise backflow along the injection tract.

### 2.4 Remote Ischemic Postconditioning and Administration of Brdu

RIPC was performed as follows. Mice under anaesthesia (1% pentobarbital sodium) had a rubber tourniquet applied tightly to the upper thigh contralateral to the brain injury for 3 min to induce limb ischemia, followed by 5 min of reperfusion upon release. This cycle of occlusion/reperfusion was repeated three times. Successful occlusion was confirmed by limb cyanosis, swelling, loss of the dorsal pedal pulse and a drop in skin temperature. To label proliferating cells, mice received an intraperitoneal injection of BrdU (50 mg/kg, HY‐15910, MCE) once daily for 5 consecutive days, starting on day 2 after MCAO. Animals were allowed to survive for an additional period (until day 28) to enable the maturation of newborn neurons before tissue collection and analysis.

### 2.5 Western Blot Analysis

Brain tissues were collected and homogenised in radioimmunoprecipitation assay (RIPA) lysis buffer to extract total protein. The protein lysates were separated by sodium dodecyl sulfate–polyacrylamide gel electrophoresis (SDS‐PAGE) and subsequently transferred onto polyvinylidene fluoride (PVDF) membranes. The membranes were then blocked and incubated overnight at 4°C with the following primary antibodies diluted in TBST: anti‐NTN4 (Rabbit, 1:1000, Huabio, ER1913‐66), anti‐CD31 (Rabbit, 1:2000, Proteintech, 28083‐1‐AP), anti‐HIF‐1α (Mouse, 1:1000, Santa Cruz, sc‐12151), anti‐VEGFA (Rabbit, 1:2000, Proteintech, 26157‐1‐AP), anti‐β‐actin (Mouse, 1:10,000, Proteintech, 66009–1‐Ig) and anti‐Lamin B1 (Rabbit, 1:10,000, Proteintech, 12987‐1‐AP), anti‐itgβ1(Rabbit，1:10000，Proteintech，12594–1‐AP)，anti‐PI3K(Rabbit，1:500，Proteintech，20584‐1‐AP)，p‐PI3K(Rabbit，1:1000，Cell Signalling technology，4228) anti‐AKT(Rabbit，1:5000，Proteintech，10176‐2‐AP)，anti‐p‐AKT(Mouce，1:5000，Proteintech，66444‐1‐Ig)，anti‐β‐actin (Mouse, 1:10,000, Proteintech, 66009‐1‐Ig)，anti‐MEK1/2(Rabbit，1:10000，Proteintech，11049‐1‐AP)，anti‐p‐MEK(Rabbit，1:1000，Cell Signalling technology，9121)，anti‐ERK1/2(Mouce，1:10000，Proteintech， 60929‐1‐Ig)，anti‐p‐ERK1/2(Rabbit，1:5000，Proteintech，80031–1‐RR). After washing, the membranes were incubated with appropriate fluorescent secondary antibodies. β‐Actin served as the loading control. Protein bands were visualised using an Odyssey infrared imaging system (LI‐COR Biosciences, Lincoln, NE, USA), and their densities were quantified using ImageJ software (National Institutes of Health, version 1.52).

### 2.6 Immunofluorescence Staining

For immunofluorescence staining, mice under deep anaesthesia were transcardially perfused with ice‐cold saline followed by 4% paraformaldehyde (PFA) in phosphate buffer. The splenium of the cortex was dissected, post‐fixed in 4% PFA at 4°C for 12 h, and then cryo‐protected in 30% sucrose solution at 4°C for 48–72 h. Tissues were sectioned coronally at a thickness of 20 μm using a cryostat, and a systematic series of 30 sections per sample was collected. Sections were incubated overnight at 4°C with the following primary antibodies: anti‐CD31 (Rabbit, 1:500, Proteintech, 28083‐1‐AP), anti‐BrdU (Mouse, 1:400, Proteintech, 66241‐1‐Ig), anti‐NeuN (Rabbit, 1:500, Proteintech, 26975‐1‐AP), anti‐NTN4 (Mouse, 1:400, R&D Systems, AF1132), anti‐βIII‐tubulin (Mouse, 1:500, Santa Cruz, sc‐51670) and anti‐GFAP (Rabbit, 1:1000, Abcam, ab7260). After washing, sections were incubated with corresponding fluorescent secondary antibodies (Goat Anti‐Mouse/Rabbit IgG Dylight 488 or 594, Abbkine). Nuclei were counterstained with DAPI (Beyotime Biotechnology). Images were acquired using a Zeiss Axioskop 40 fluorescence microscope. The mean fluorescence intensity was quantified semi‐automatically using Image‐Pro Plus 6.0 software.

### 2.7 Behavioural Test

Spatial learning and memory were assessed using the Morris water maze. Mice were trained to locate a submerged, hidden platform (1.5 cm below the water surface) in a circular pool. Training consisted of four trials per day for five consecutive days. During each trial, mice were allowed to swim until they found the platform or until 60 s elapsed, at which point they were guided to it and remained for 15 s. On the sixth day, a probe trial was conducted with the platform removed; mice swam freely for 60 s. A video tracking system recorded the escape latency during training and the number of platform crossings during the probe trial.

General locomotor activity and anxiety‐like behaviour were evaluated in an open‐field arena (50 × 50 × 40 cm). Each mouse was gently placed in the same corner and allowed to explore freely for 30 min after a 1‐min habituation period. Movements were recorded by an overhead camera, and the total distance travelled, number of entries into the central zone and time spent in the centre were analysed using automated tracking software.

### 2.8 Isolation and Culture of Primary Mouse Brain Microvascular Endothelial Cells

Primary mouse brain microvascular endothelial cells (BMECs) were isolated from postnatal day 7 (P7) C57BL/6 mice. Under deep anaesthesia (sodium pentobarbital, 50 mg/kg), cerebral cortices were dissected, minced into approximately 1‐mm^3^ fragments and subjected to enzymatic digestion. The tissue fragments were digested in 0.1% trypsin–EDTA (Gibco) at 37°C for 15 min, with gentle vortexing every 3 min to ensure uniform dissociation. The digestion was stopped by adding an equal volume of high‐glucose DMEM (HyClone) containing 20% fetal bovine serum (FBS, Gibco). The cell suspension was then mixed with three volumes of 30% bovine serum albumin (BSA) and centrifuged at 1500 rpm for 5 min. The resulting pellet was resuspended in complete endothelial cell growth medium (ECGM, ScienCell) and seeded into L‐polylysine‐coated T25 culture flasks. Cells were maintained at 37°C in a humidified incubator with 5% CO_2_.

### 2.9 Extraction and Culture of Primary Neural Stem Cells

Primary neural stem cells (NSCs) were isolated from the cerebral cortices of postnatal day 1–3 (P1–P3) C57BL/6 mouse pups, as previously described with modifications. Briefly, pups were euthanised, and the heads were surface‐sterilised in 75% ethanol for approximately 10 s. Cortical tissues were dissected under a stereomicroscope and minced into approximately 1 mm^3^ fragments. Tissue fragments were enzymatically digested at 37°C for 20 min in a solution containing papain (MERCK, 76216) and DNase I (MERCK, ZYMW1001), with gentle trituration performed 8–10 times every 5 min. The digestion was stopped by adding an equal volume of DMEM/F‐12 supplemented with 10% fetal bovine serum. The cell suspension was centrifuged at 500 rpm for 5 min, and the pellet was resuspended in proliferation medium consisting of Neurobasal Medium (ThermoFisher, 21103049) supplemented with 2% B27 (ThermoFisher, 17504044), 1 mM GlutaMAX, 10 ng/mL basic fibroblast growth factor (PeproTech, 450–33), 10 ng/mL epidermal growth factor (PeproTech, 315–09) and 1% penicillin–streptomycin (ThermoFisher, 15140122). The suspension was passed through a 40 μm cell strainer to obtain a single‐cell suspension. Isolated NSCs were then plated in T75 flasks for expansion in suspension culture using the same proliferation medium.

### 2.10 Lentiviral Transfection

Isolated BMECs were seeded into 24‐well plates at a density of 5 × 10^4^ cells per well and pre‐cultured in serum‐free medium for 24 h. Upon reaching approximately 50% confluence, cells were transduced with high‐titre lentiviral particles (LV‐oe*Ntn4*‐GFP; Shanghai Genechem Co. Ltd) carrying the full‐length mouse *Ntn4* cDNA under the control of the CMV promoter. The viral stock had a titre of 2 × 10^8^ TU/mL as determined by Lenti‐X GoStix. Cells were infected at a multiplicity of infection (MOI) of 5 in fresh medium supplemented with 8 μg/mL polybrene (hexadimethrine bromide) for 24 h at 37°C under 5% CO_2_. The viral supernatant was then replaced with complete growth medium. Control cells were transduced with an empty vector lentivirus of identical backbone under the same conditions. Transduction efficiency, assessed by the percentage of GFP‐positive cells 72 h post‐infection, exceeded 85%.

### 2.11 Small Interfering RNA Transfection

Primary BMECs and NSCs were seeded separately in 12‐well plates at a density of 5 × 10^4^ cells per cm^2^ in Opti‐MEM reduced serum medium (Gibco, 31985070). For siRNA transfection, 25 nM of integrin β1‐targeting siRNA was mixed with 0.5 μL of Lipofectamine RNAiMAX transfection reagent (Invitrogen, 13778030) in 100 μL of Opti‐MEM. The mixture was incubated at room temperature for 20 min to allow complex formation, then added dropwise to the cells. Following a 6‐h incubation at 37°C under 5% CO_2_, the transfection mixture was replaced with normal growth medium.

### 2.12 Tube Forming Assay in Vitro

To assess in vitro angiogenic capacity, primary mouse BMECs were seeded onto growth factor‐reduced Matrigel‐coated 24‐well plates and cultured for 12 h at 37°C under 5% CO_2_ in 1.5 mL of serum‐free DMEM supplemented with recombinant NTN‐4 protein. Tube formation was subsequently visualised and imaged using an inverted fluorescence microscope (Carl Zeiss, Oberkochen, Germany).

### 2.13 Molecular Docking Analysis

The three‐dimensional structure of the target protein was retrieved from the RCSB PDB or AlphaFold2 database. Prior to docking, the structure was preprocessed using PyMOL v2.5.2 to remove water molecules, model missing residues with the Mutagenesis Wizard, and standardise chain identifiers. Active and passive residues for docking were defined based on prior literature or predicted using CPORT. Protein–DNA docking was performed using the HADDOCK v2.4 web server in high‐resolution sampling mode with unambiguous restraints, generating 1000 initial complex structures that were subsequently clustered by HADDOCK score. The top‐ranked cluster representative was analysed in PyMOL to identify key intermolecular interactions, including hydrogen bonds (donor‐acceptor distance < 3.5 Å), salt bridges (< 4.0 Å) and hydrophobic contacts. The binding affinity (predicted Kd) was estimated using PRODIGY. Finally, the stability of the docked complex was assessed through 100‐ns molecular dynamics simulations performed with GROMACS 2022.4 (AMBER99SB‐ILDN force field). The binding free energy (ΔG) was calculated using the MM/PBSA method on the last 20 ns of the trajectory.

### 2.14 DNA Pulldown Assay

Nuclear extracts (200 μg) isolated from the brains of RIPC‐treated MCAO mice were incubated with 2 pmol of biotinylated double‐stranded DNA probes containing the predicted HIF‐1α binding motif (5′‐GACGTGC‐3′) from the *Ntn4* promoter. Incubation was carried out for 1 h at 4°C in binding buffer (10 mM Tris–HCl, pH 7.5, 50 mM KCl, 1 mM DTT) containing 1 μg of poly(dI:dC) as a nonspecific competitor. Streptavidin‐conjugated magnetic beads were then added to capture the DNA‐protein complexes. After three washes with a high‐stringency buffer (0.1% NP‐40, 500 mM NaCl), bound proteins were eluted by heating at 95°C for 10 min in 2× SDS loading buffer. HIF‐1α binding was detected by immunoblotting using a specific antibody (Cell Signalling, #14179, 1:1000). Control experiments were performed using a scrambled sequence probe and a mutant probe (GACGTGC→AAATTA). Binding enrichment was quantified by comparing the band intensity from the pulldown sample to 10% of the input nuclear extract.

### 2.15 ChIP‐PCR

Brain tissues were cross‐linked with 1% formaldehyde and quenched with glycine. Chromatin was sheared to 200–500 bp fragments (Covaris S220). Immunoprecipitation was performed overnight at 4°C using an anti‐HIF‐1α antibody (5 μg) or control IgG with Protein A/G magnetic beads. After washing and reverse cross‐linking, precipitated DNA was analysed by qPCR with primers targeting the *Ntn4* enhancer. Results were normalised to 10% input DNA.

### 2.16 Statistical Analysis

All quantitative data are presented as the mean ± standard error of the mean (SEM). Statistical analyses were conducted using GraphPad Prism 7.0 (GraphPad Software, La Jolla, CA, USA). For comparisons among multiple groups, one‐way analysis of variance (ANOVA) was applied, followed by Tukey's honestly significant difference (HSD) post hoc test for pairwise comparisons when the overall ANOVA was significant. Data distribution was assumed to be normal, and variance was assumed to be similar between groups. A two‐tailed *p*‐value of < 0.05 was considered statistically significant. To minimise bias, all image quantification and behavioural scoring were performed by investigators blinded to the experimental groups.

## 3 Results

### 3.1 RIPC Upregulates Cerebral NTN4 Expression in MCAO Mice

**FIGURE 1:**
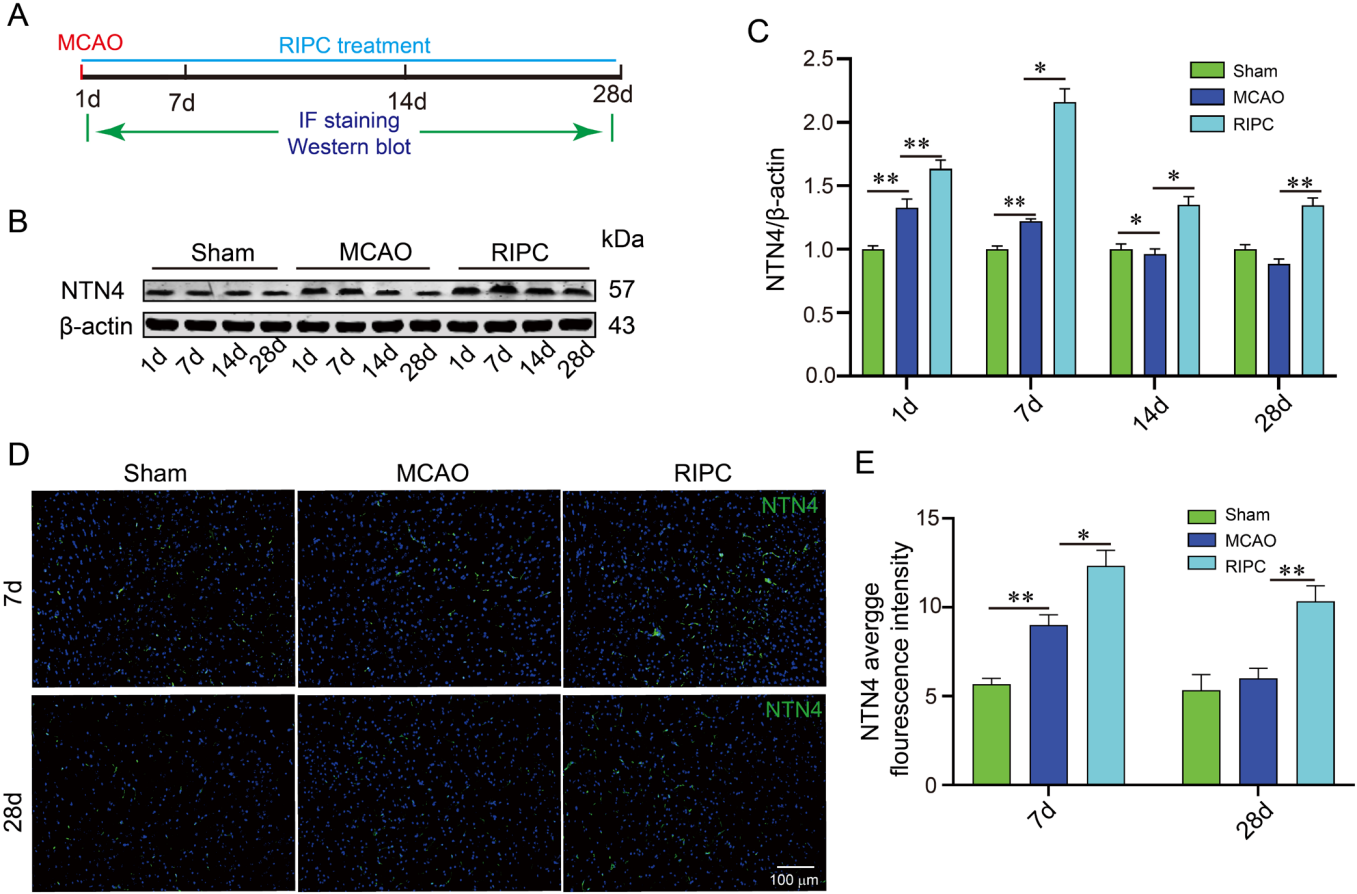
RIPC treatment upregulates netrin‐4 expression in MCAO mice. (A) Schematic of MCAO modelling and tissue collection timeline. (B, C) Western blots and densitometric quantification of NTN4 expression normalised to β‐Actin. (D, E) Immunofluorescence staining and mean fluorescence intensity (MFI) of NTN4 (green) in peri‐infarct cortex. Cell nuclei counterstained with DAPI (blue). Bars = 100 μm. *p < 0.05, **p < 0.01 (*n* = 4 biologically independent animals per group).

The MCAO model was established and subjected to RIPC. Brain tissue samples were collected at 1, 7, 14 and 28 days after MCAO (Figure 1A). Initial experiments confirmed that RIPC enhanced angiogenesis (Figure S1A,B,D) and neurogenesis (Figure S1A,C) while alleviating ischemia‐induced neuronal damage (Figure S2) in MCAO mice. Interestingly, western blot analysis showed that NTN4 expression was significantly increased in the brains of MCAO mice compared with the sham group at 1 and 7 days post‐MCAO, but markedly decreased at 14 and 28 days (Figure 1B,C). In contrast, RIPC treatment significantly upregulated NTN4 protein levels at all time points relative to the MCAO group (Figure 1B,C). Immunofluorescence staining of NTN4 in mouse brain tissue at 7 and 14 days yielded results consistent with the western blot findings (Figure 1D,E).

### 3.2 Supplementation With Recombinant NTN4 Rescues Angiogenesis and Neurogenesis in NTN4‐Deficient Mice

**FIGURE 2:**
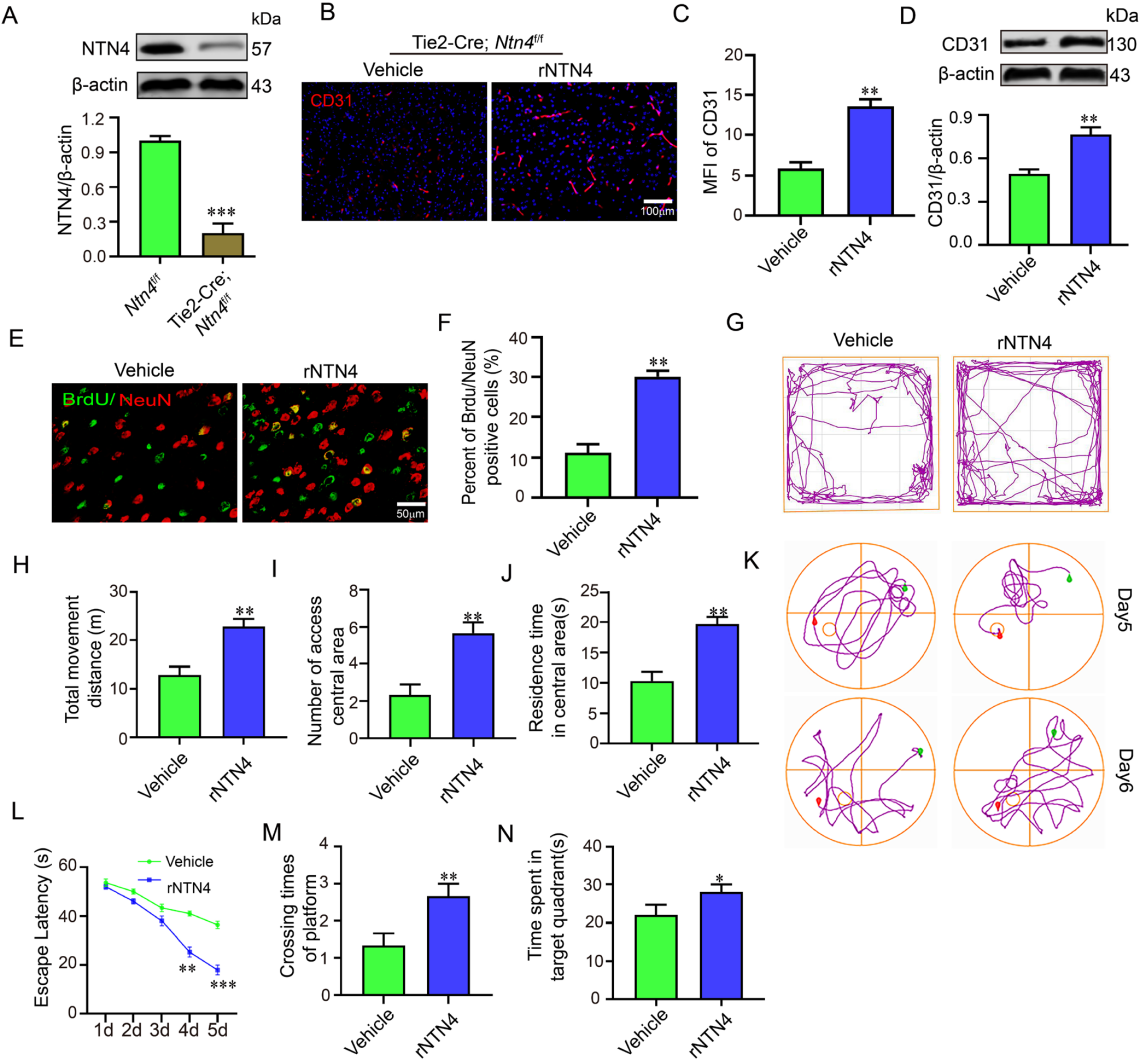
Effects of recombinant Netrin‐4 (rNTN4) treatment in endothelial‐specific NTN4 knockout mice (Tie2‐Cre; *NTN4* ^*f/f*^). (A) Western blot detecting NTN4 protein expression to evaluate knockout efficiency. (B, C) Representative images of CD31‐immunostained microvessels (red) in the peri‐infarct cortex and corresponding quantitative analysis. (D) CD31 protein levels measured by western blotting. (E, F) BrdU^+^/NeuN^+^ co‐staining (green/red) identifying newborn neurons and quantitative analysis of newborn neuron counts. (G–J) Open field test: Locomotor trajectory maps, total movement distance, number of centre zone crossings, time spent in the centre zone. (K) Representative swimming trajectory maps in the Morris water maze test. (L) Latency to locate the hidden platform during the training phase. (M, N) Number of platform crossings within 1 min after platform removal and time spent in the target quadrant during probe trial. *p < 0.05, **p < 0.01, ***p < 0.001 (*n* = 8 biologically independent animals per group).

To determine whether NTN4 contributes to the therapeutic effects of RIPC in stroke, we generated endothelial‐specific NTN4 conditional knockout mice (Tie2‐Cre; *Ntn4* ^f/f^). Western blot analysis confirmed a significant reduction in NTN4 protein in Tie2‐Cre; *Ntn4* ^f/f^ mice compared with *Ntn4* ^f/f^ controls (Figure 2A), verifying successful knockout. Supplementation with recombinant NTN4 increased the mean fluorescence intensity (MFI) of CD31, an endothelial cell marker and the protein level of CD31 (Figure 2B–D). Co‐labelling BrdU (a proliferation marker) and NeuN (a neuronal marker) revealed that the percentage of BrdU/NeuN double‐positive cells was higher in the rNTN4‐treated group than the vehicle group (Figure 2E,F). In the open field test, rNTN4‐treated mice exhibited increased total movement distance, more entries into the central area and longer residence time in the centre compared with vehicle‐treated mice (Figure 2G–J). Morris water maze performance was assessed by escape latency, number of platform crossings and time spent in the target quadrant. rNTN4‐treated mice showed shorter escape latency, crossed the former platform location more frequently and spent significantly more time in the target quadrant than vehicle‐treated controls (Figure 2K–N).

### 3.3 RIPC Improves Neurological Function After Stroke via NTN4 Activation

**FIGURE 3:**
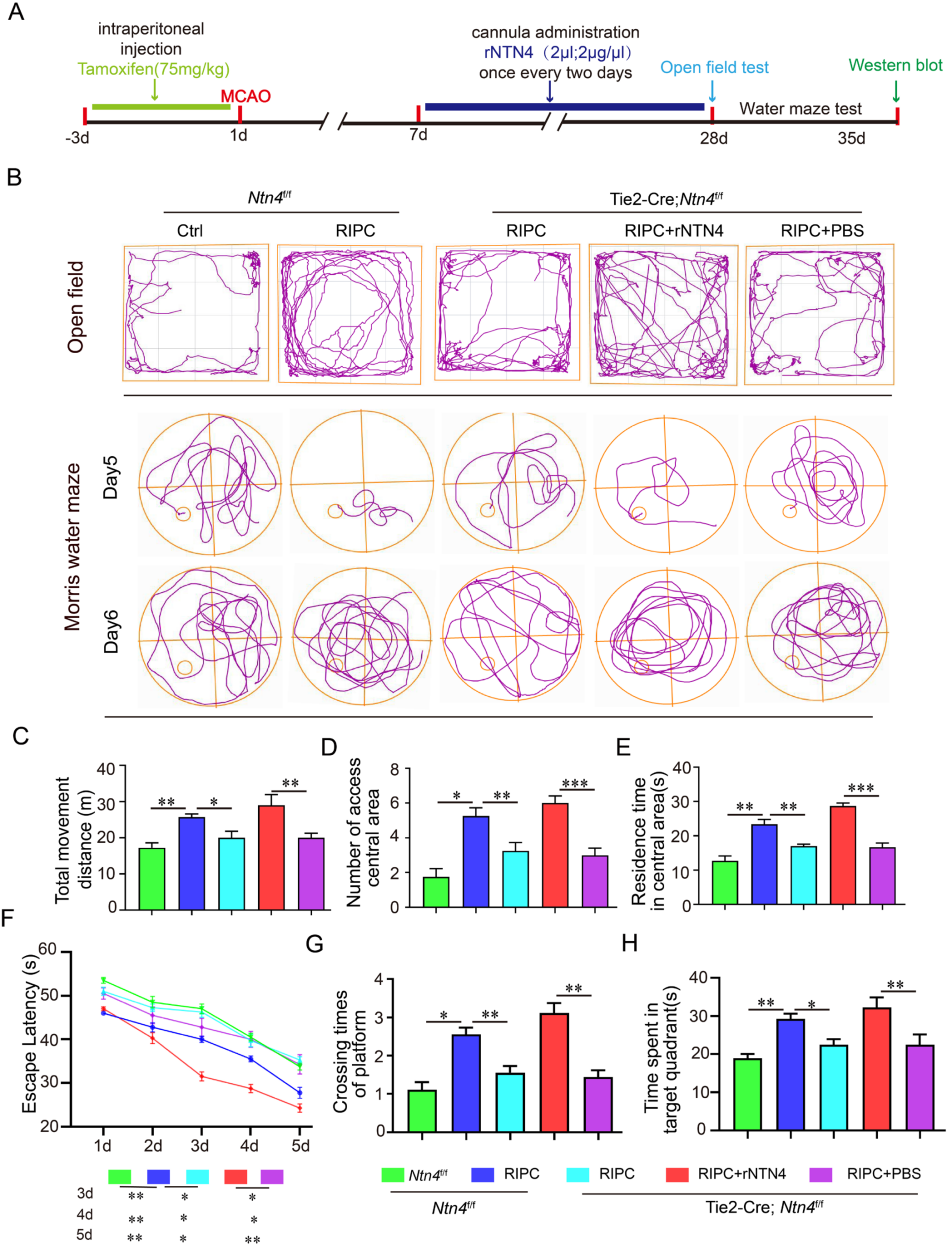
RIPC improves neurological function after stroke via NTN4 activation. (A) Experimental workflow. Endothelial‐specific NTN4 KO mice (Tie2‐Cre; *NTN4* ^*f/f*^) received rNtn4 at 7d post‐MCAO. (B, C) Representative trajectory maps of mice in open field and water maze experiments. (D–F) The open field test assessed the total movement distance, number of centre zone crossings, and duration spent in the centre zone. (G) Latency to locate the hidden platform during the training phase. (H, I) Number of platform crossings within 1 min after platform removal and time spent in the target quadrant during the probe trial. *p < 0.05, **p < 0.01, ***p < 0.001 (*n* = 8 biologically independent animals per group).

All experimental groups underwent MCAO. The Tie2‐Cre; *Ntn4* ^f/f^ + rNtn4 group received an intracerebral injection of recombinant NTN4 (rNtn4) 7 days after MCAO, and behavioural assessments were performed at 28 days (Figure 3A). In the open field test, *Ntn4* ^f/f^ + RIPC mice showed longer travel distances, more central‐zone crossings and increased time spent in the central zone than *Ntn4* ^f/f^ controls (Figure 3B–E). Although Tie2‐Cre; *Ntn4* ^f/f^ + RIPC mice displayed a modest increase in these parameters relative to Tie2‐Cre; *Ntn4* ^f/f^ mice, their performance remained significantly lower than that of *Ntn4* ^f/f^ + RIPC mice. Similarly, in the water maze test, *Ntn4* ^f/f^ + RIPC mice had significantly shorter escape latencies, more platform crossings and longer dwell times in the target quadrant than Tie2‐Cre; *Ntn4* ^f/f^ + RIPC mice (Figure 2C,G–I). Together, these results indicate that endothelial‐specific deletion of NTN4 attenuates the ability of RIPC to promote neurological recovery after MCAO.

### 3.4 RIPC Promotes Angiogenesis and Neurogenesis After MCAO via NTN4

**FIGURE 4:**
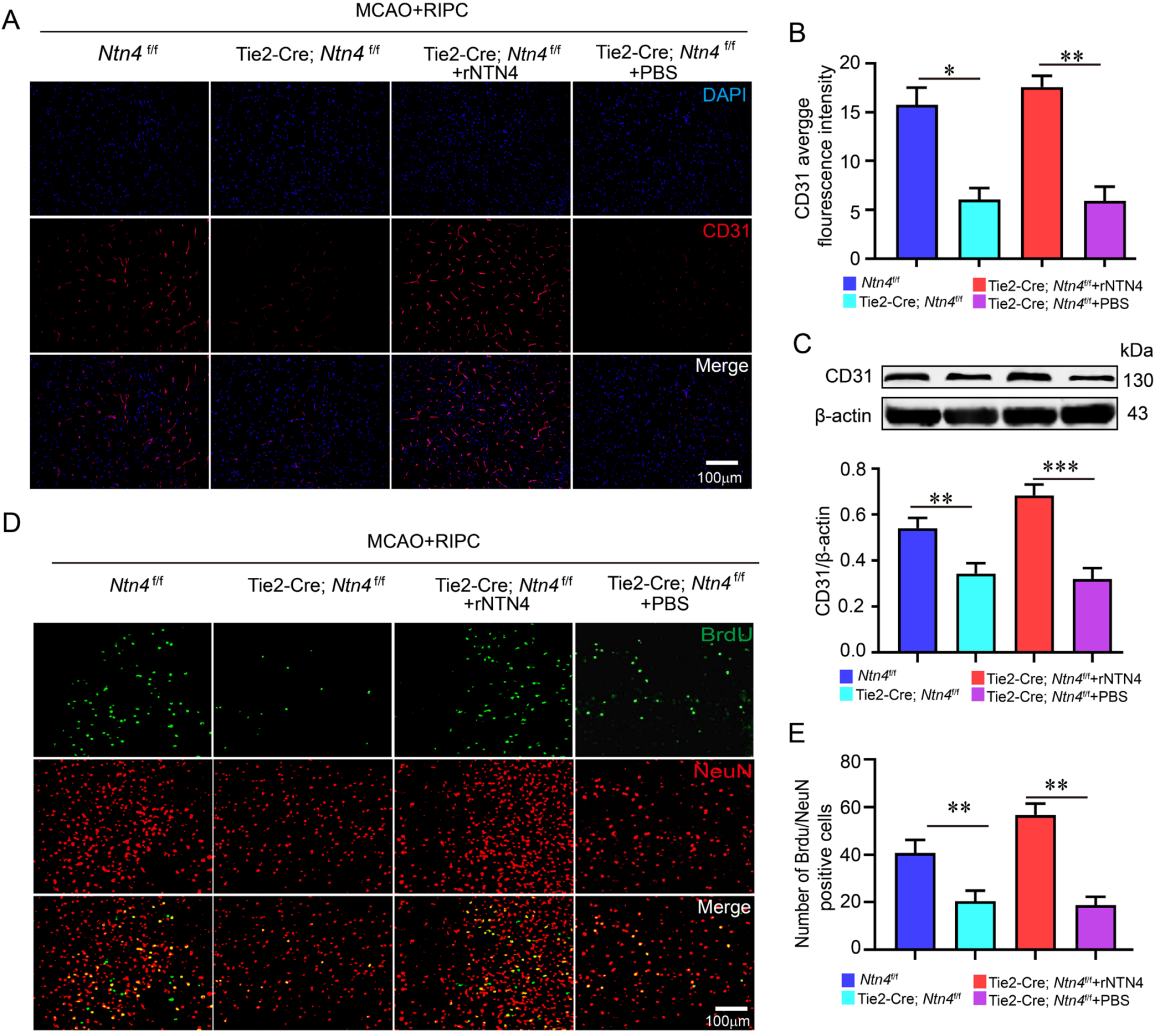
RIPC promotes angiogenesis and neurogenesis after MCAO via NTN4. (A, B) Cortical microvessels were visualised by CD31 immunostaining (red), with representative images shown and vessel density quantified. (C) CD31 protein expression levels were determined by western blot analysis. (D, E) Newborn neurons were identified by BrdU^+^/NeuN^+^ co‐staining (green/red) and subsequently quantified. Bars = 100 μm. *p < 0.05, **p < 0.01, ***p < 0.001 (*n* = 4 biologically independent animals per group).

While the beneficial effects of RIPC on angiogenesis and neurogenesis in MCAO mice have been reported, the underlying mechanisms are not fully understood. Immunofluorescence staining for the endothelial marker CD31 showed that the fluorescence intensity of cerebral microvessels in Tie2‐Cre; *Ntn4* ^f/f^ mice was significantly lower than in *Ntn4* ^f/f^ mice following RIPC. This reduction was reversed by exogenous administration of recombinant NTN4 protein (Figure 4A,B), a result further confirmed by western blot analysis (Figure 4C). To evaluate neurogenesis, BrdU and NeuN co‐immunostaining were performed. The number of newborn neurons (BrdU^+^/NeuN^+^ cells) was markedly lower in *Ntn4*‐knockout mice treated with RIPC compared with *Ntn4* ^f/f^ controls. Notably, supplementing recombinant NTN4 protein restored the impaired neurogenesis resulting from NTN4 deficiency (Figure 4D,E). Collectively, these results demonstrate that NTN4 is essential for RIPC‐induced angiogenesis and neurogenesis after cerebral ischemia.

### 3.5 NTN4 Promotes Endothelial Cell Proliferation and Angiogenesis

**FIGURE 5:**
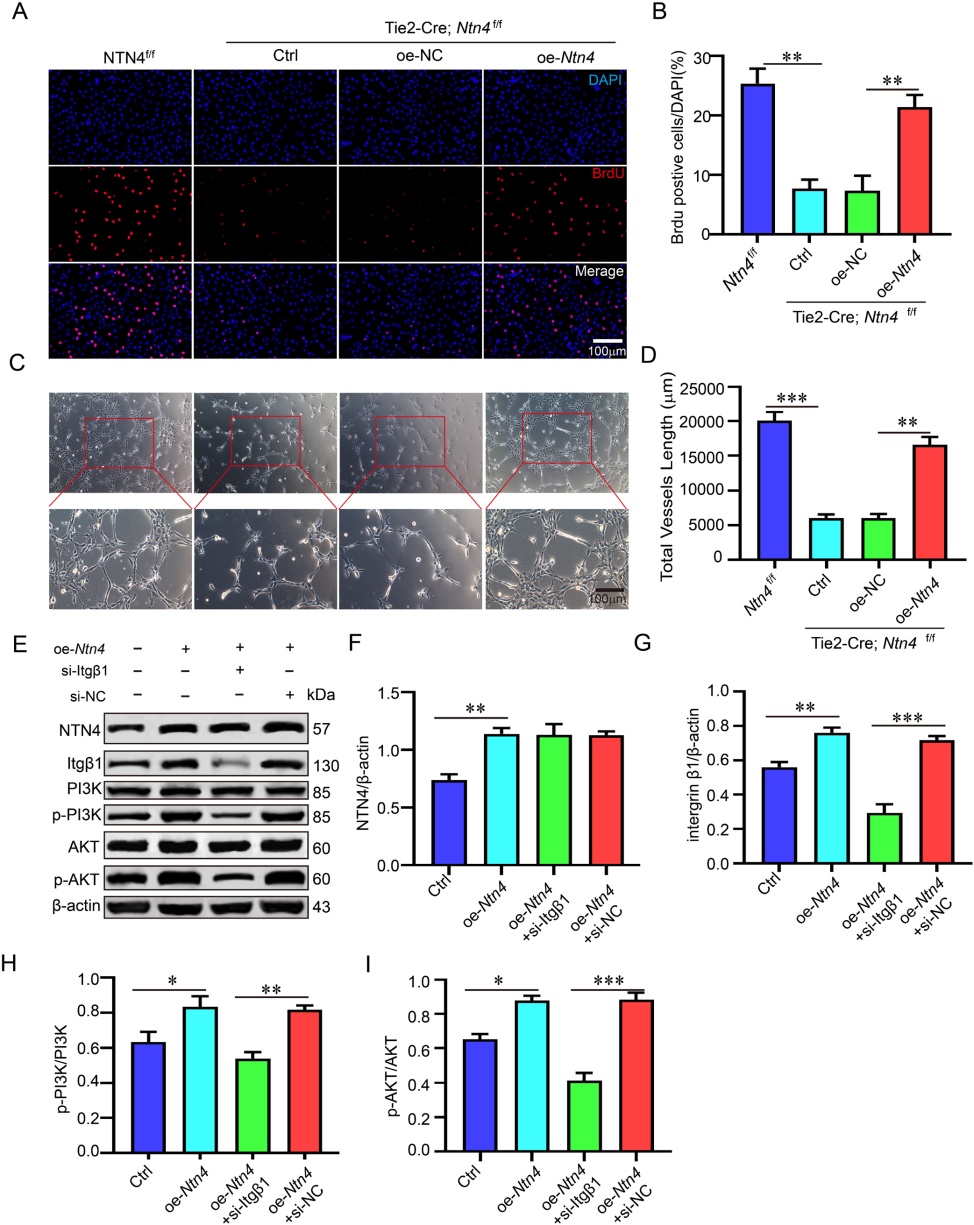
NTN4 promotes endothelial cell proliferation and angiogenesis. (A, B) Primary BMEC proliferation was evaluated using a BrdU incorporation assay (BrdU: Red, nuclei: DAPI blue) and quantified by counting BrdU^+^ cells. (C, D) Angiogenic capacity was analysed by tube formation assay, and the total network length was measured. (E–I) Protein levels of key components in the PI3K/AKT pathway were analysed by western blotting after Itgβ1 knockdown and oe‐NTN4 treatment. Bars = 100 μm. *p < 0.05, **p < 0.01, ***p < 0.001 (*n* = 4 biologically independent animals per group).

Primary BMECs were isolated from *Ntn4* ^f/f^ and Tie2‐Cre; *Ntn4* ^f/f^ mice. After incubation with BrdU (0.03 μg/mL) for 40 min, immunofluorescence staining was performed to assess cell proliferation. Results showed that the proliferative capacity of endothelial cells was significantly lower in the Tie2‐Cre; *Ntn4* ^f/f^ group than in the *Ntn4* ^f/f^ group, while *Ntn4* overexpression reversed this effect (Figure 5A,B). Tube‐formation assays revealed that total vascular network length was markedly reduced in the Tie2‐Cre; *Ntn4* ^f/f^ group compared with the *Ntn4* ^f/f^ group. Conversely, *Ntn4* overexpression significantly enhanced the tube‐forming ability of endothelial cells (Figure 5C,D). To elucidate the downstream pathway underlying NTN4‐induced angiogenesis, integrin β1 was knocked down using siRNA in *Ntn4*‐overexpressing cells. The results demonstrated that *Ntn4* overexpression increased integrin β1 protein levels and activated the PI3K/AKT signalling pathway (Figure 5E–I).

### 3.6 NTN4 Promotes the Differentiation of NSCs Into Neurons

**FIGURE 6:**
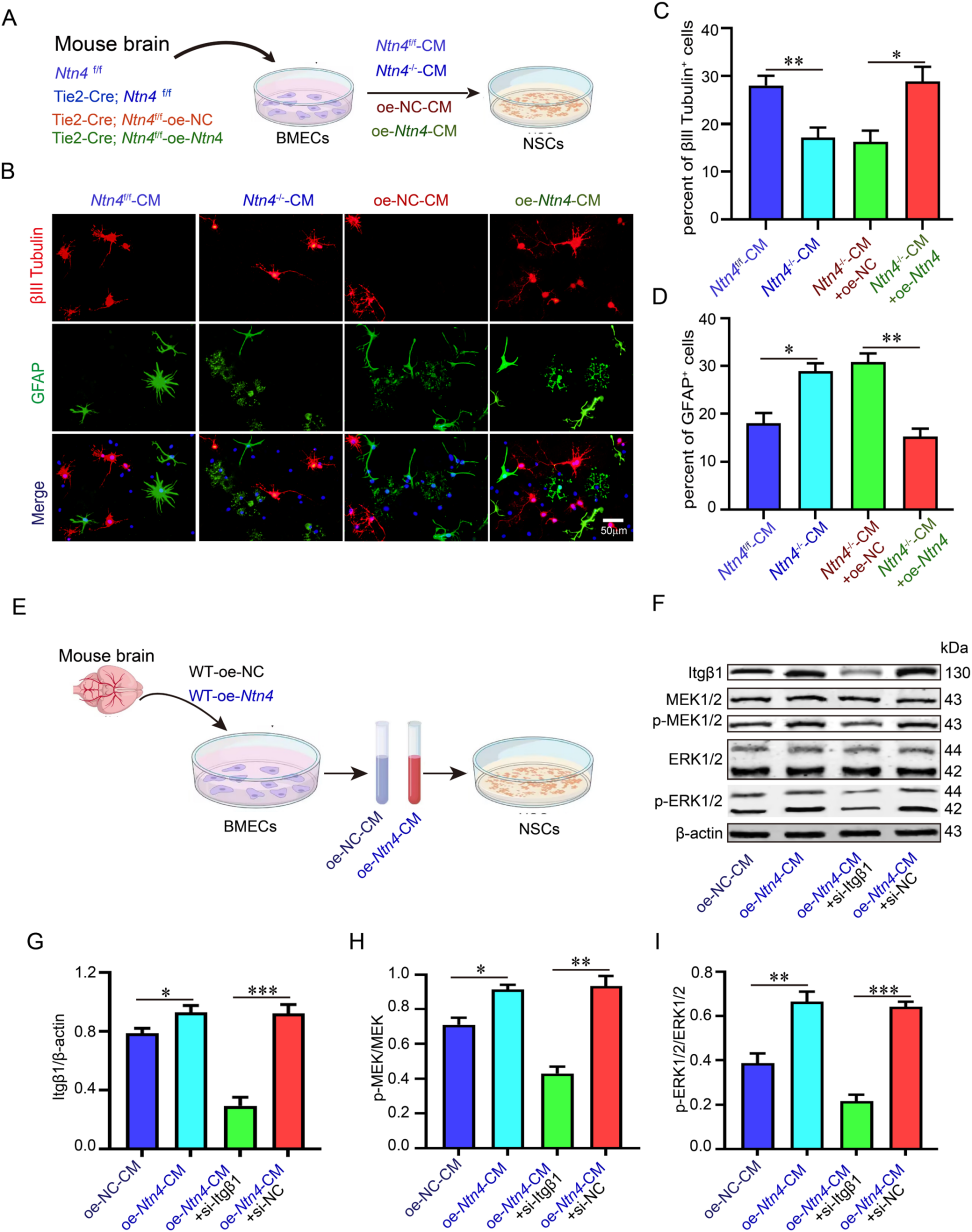
NTN4 promotes the differentiation of NSCs into neurons. (A) A schematic diagram illustrates the treatment of neural stem cells (NSCs) with primary BMEC‐conditioned medium (CM) from four groups: NTN^f/f^‐CM, NTN^−/−^‐CM, oeNC‐CM and oeNTN4‐CM. (B) Cell differentiation was assessed by immunofluorescence for the neuronal marker βIII‐tubulin (red) and the astrocytic marker GFAP (green), with nuclei counterstained by DAPI (blue). (C, D) The percentages of βIII‐tubulin^+^ neurons and GFAP^+^ astrocytes were quantified. (E) A schematic summarises the proposed signalling mechanism of oeNTN4‐CM on NSCs with Itgβ1 knockdown. (F–I) To investigate the involved pathway, the expression of MAPK/ERK‐related proteins was analysed by western blotting after Itgβ1 knockdown. Bars = 100 μm. *p < 0.05, **p < 0.01, ***p < 0.001 (*n* = 4 biologically independent animals per group).

To examine the effect of endothelial‐derived NTN4 on NSCs differentiation, primary NSCs were isolated from wild‐type (WT) mice and treated with conditioned medium (CM) collected from BMECs (Figure 6A). Immunofluorescence staining was performed for the neuronal marker βIII‐tubulin and the astrocytic marker glial fibrillary acidic protein (GFAP). Compared with the CM from *Ntn4* ^f/f^ endothelial cells (*Ntn4* ^f/f^‐CM), CM from Tie2‐Cre; *Ntn4* ^f/f^ endothelial cells (*Ntn4* ^−/−^‐CM) significantly increased the percent of GFAP‐positive cells and decreased the percent of βIII‐tubulin‐positive cells (Figure 6B–D). In contrast, CM from *Ntn4*‐overexpressing endothelial cells (oe*Ntn4*‐CM) reduced the percent of GFAP‐positive cells relative to CM from control‐transfected endothelial cells (oeNC‐CM) (Figure 6B,D), while markedly increasing the number of βIII‐tubulin‐positive cells (Figure 6B,C). To determine whether NTN4 promotes neuronal differentiation of NSCs by activating the MAPK/ERK pathway via integrin β1, integrin β1 was knocked down in NSCs, which were then cultured with CM from *Ntn4*‐overexpressing endothelial cells. Western blot analysis showed that the oe*Ntn4*‐CM group exhibited elevated integrin β1 protein levels and significantly increased phosphorylation of MEK1/2 and ERK1/2 compared with the oeNC‐CM group; however, integrin β1 knockdown reversed these effects (Figure 6E–H).

### 3.7 RIPC Activates NTN4 Transcription via Upregulation of HIF‐1α

**FIGURE 7:**
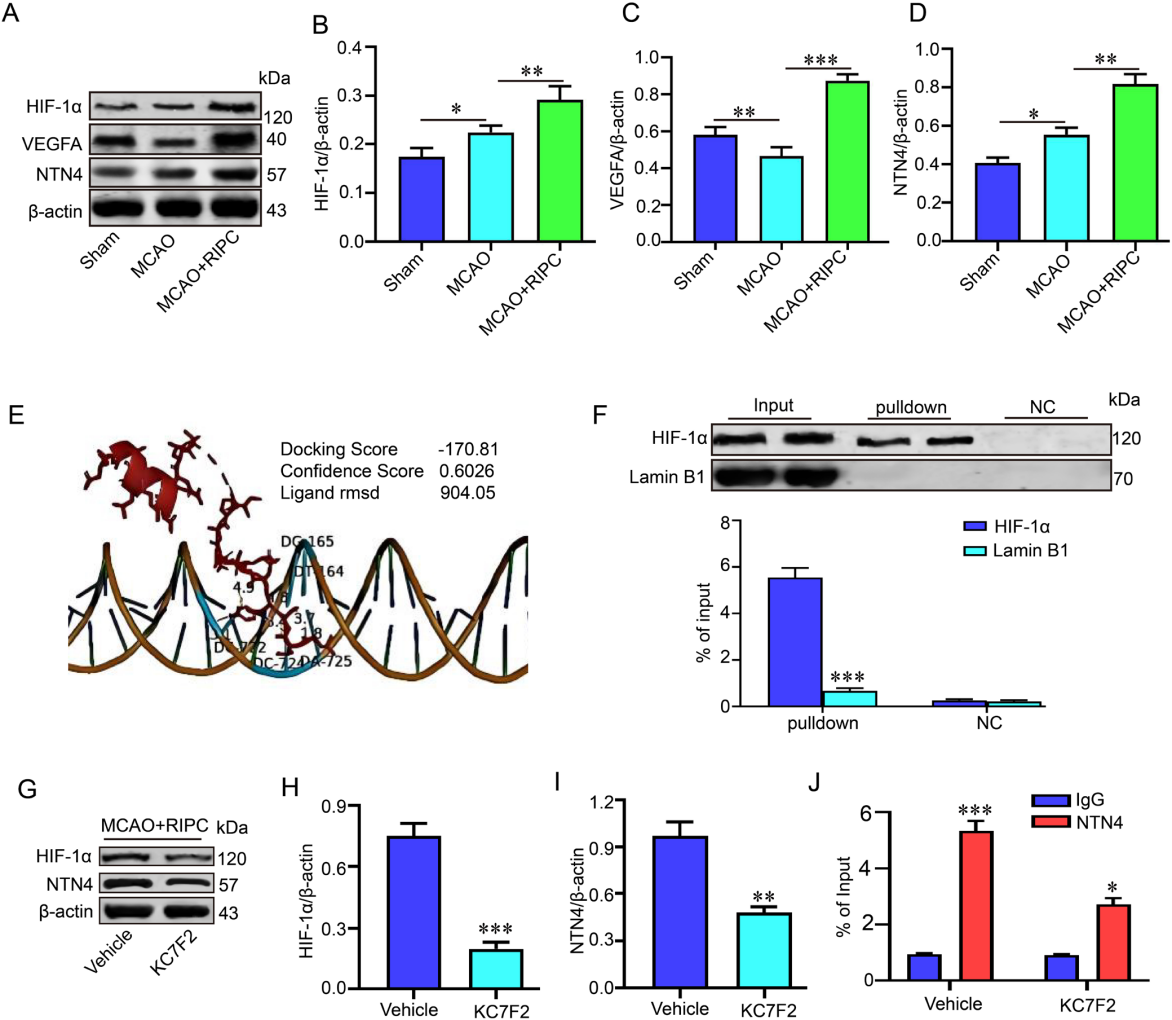
RIPC activates NTN4 transcription via upregulation of HIF‐1α. (A–D) Protein expression levels of HIF‐1α, VEGFA and NTN4 were assessed by western blotting and quantified by densitometric analysis. (E) A molecular docking model predicted the interaction between HIF‐1α (red) and the NTN4 promoter DNA sequence (orange), with an HDOCK score of −170.81. (F) Direct binding of HIF‐1α to the NTN4 promoter was verified by a DNA pulldown assay. (G, I) Representative western blots and quantification of HIF‐1α and NTN4 proteins. (J) ChIP assay demonstrated specific enrichment of the NTN4 promoter region by HIF‐1α. *p < 0.05, **p < 0.01, ***p < 0.001 (*n* = 3 biologically independent animals per group).

To explore how RIPC regulates NTN4 expression, we first examined protein levels in the brains of MCAO mice. RIPC treatment significantly increased the expression of HIF‐1α, VEGFA and NTN4 compared with the MCAO group (Figure 7A–D). Given that the HIF‐1α/VEGFA pathway is a well‐established regulator of angiogenesis and considering previous reports that HIF‐1α transcriptionally controls NTN1—a protein structurally similar to NTN4—we hypothesised that HIF‐1α might also bind to the *Ntn4* promoter. Molecular docking using HDOCK software predicted stable binding between HIF‐1α and the *Ntn4* promoter region (Figure 7E). This interaction was confirmed experimentally by DNA pulldown assay, which showed strong enrichment of HIF‐1α protein on an *Ntn4* promoter‐specific probe (Figure 7F). To further validate the HIF‐1α‐dependent transcription of *Ntn4*, we inhibited HIF‐1α using KC7F2 (an established HIF‐1α inhibitor) in MCAO mice treated with RIPC. Western blot analysis showed that NTN4 protein expression was significantly lower in the KC7F2‐treated group compared to the vehicle control group (Figure 7G–I). Correspondingly, ChIP assays revealed a marked reduction in the enrichment of DNA fragments containing the HIF‐1α‐binding motif within the endogenous *Ntn4* promoter following KC7F2 treatment (Figure 7J). These results confirm that HIF‐1α directly binds to the *Ntn4* promoter in vivo and is functionally required for its transcriptional activation. Taken together, these results indicate that RIPC upregulates HIF‐1α, which in turn binds directly to the *Ntn4* promoter and activates its transcription, leading to increased NTN4 protein expression.

## 4 Discussion

Clinically, RIPC induces transient limb ischemia to confer neuroprotection and has well‐documented efficacy [23, 24]. Mechanistically, it is known to promote neuronal survival by suppressing endoplasmic reticulum stress and inhibiting p38/MAPK signalling, and to regulate neuroinflammation via Treg‐mediated microglial control [25, 26]. While these pathways explain aspects of cellular protection, a critical gap remains in understanding how the systemic RIPC stimulus initiates local brain repair mechanisms, specifically angiogenesis and neurogenesis. This study elucidates a novel HIF‐1α/ NTN4 pathway essential for the protective effects of RIPC against ischemic stroke. Key findings include: (a) RIPC prevents the post‐stroke decrease in NTN4 expression; (b) NTN4is a required mediator, as endothelial‐specific knockout impaired RIPC‐induced angiogenesis and neurogenesis—effects restored by recombinant NTN4 and (c) mechanistically, RIPC enhances HIF‐1α levels, which directly activate NTN4 transcription. Collectively, these results position the HIF‐1α/ NTN4 axis as a critical mechanism for RIPC‐induced protection.

Prior studies have shown that NTN4 can suppress neuroinflammation, enhance endothelial function and improve post‐stroke recovery [15, 16], consistent with our in vitro pro‐angiogenic and neurogenic observations. However, other reports suggest NTN4 may inhibit angiogenesis, possibly by disrupting laminin‐γ1/extracellular matrix interactions [27, 28, 29]. This contradiction underscores the context‐dependent nature of NTN4 signalling. Using KO mice and recombinant protein rescue, we established that endothelial NTN4 is both necessary and sufficient for RIPC‐mediated vascular and neural repair, defining it as a positive regulator in this setting. While NTN4 promotes endothelial and NSCs responses, the specific receptors and downstream pathways involved remain unclear. To address this, we focused on integrin α6β1, a known functional NTN4 receptor in both mouse NSCs [30] and endothelial cells [31]. Given the established link between integrin β1 (Itgβ1) and the pro‐angiogenic PI3K/AKT pathway [32, 33], we hypothesised that NTN4 acts through Itgβ1 to activate key signalling cascades. Testing this hypothesis revealed that Itgβ1 knockdown reversed the activation of the PI3K/AKT pathway induced by *Ntn4* overexpression in endothelial cells, indicating that *Ntn4* promotes angiogenesis via an Itgβ1‐PI3K/AKT axis. Furthermore, considering the role of MAPK/ERK signalling in neurogenesis and evidence that Itgβ1 can activate MEK/ERK [34, 35], we investigated a parallel mechanism. We found that Itgβ1 knockdown also abolished the activation of MEK/ERK pathways induced by conditioned medium from *Ntn4*‐overexpressing endothelial cells. This confirms that *Ntn4* promotes neuronal differentiation through Itgβ1‐dependent activation of the MAPK/ERK pathway.

Mechanistically, we found that RIPC upregulates NTN4 by stabilising the transcription factor HIF‐1α, a key mediator of hypoxic responses and RIPC‐induced protection. Since HIF‐1α is known to transcriptionally activate NTN1 via hypoxia‐response elements (HREs), and given the presence of conserved HRE cores in the *Ntn4* promoter, we hypothesise that HIF‐1α directly regulates NTN4. This was initially suggested by *in silico* docking predictions and confirmed in vitro by DNA pull‐down assays. More definitively, in vivo ChIP assays demonstrated HIF‐1α enrichment on the endogenous *Ntn4* promoter in the RIPC‐treated ischemic cortex. To establish functional causality, pharmacological inhibition of HIF‐1α with KC7F2 abolished RIPC‐induced NTN4 upregulation, proving the dependence of this regulatory axis on HIF‐1α.

We acknowledge several limitations. Our findings are derived from young male mice, which constrains their predictive value for the diverse clinical stroke population and highlights a translational gap. Regarding the genetic model, although the commercially sourced and validated *Ntn4*(f/f); Tie2‐Cre lines allowed us to establish a foundational role for endothelial NTN4, Tie2 expression in haematopoietic lineages means that conditional knockout in specific sub‐lineages (e.g., using Cdh5‐CreERT2) is a necessary next step to identify the precise cellular source. Furthermore, while our previous data indicated predominant NTN4 expression in endothelial cells [22], its cellular source within the peri‐infarct milieu, including potential contributions from astrocytes or specific subpopulations, remains incompletely defined and warrants further investigation. Lastly, our conclusions on angiogenesis and neurogenesis are primarily based on marker analyses (CD31, NeuN) and tube formation assays. Future work would benefit from functional endpoint validations, such as assessing vessel perfusion and BBB integrity for angiogenesis, and employing long‐term tracing to confirm the survival and integration of newborn neurons.

In conclusion, we establish that the neurovascular protective effects of RIPC are mechanistically coordinated through the HIF‐1α/NTN4 signalling axis. We identify NTN4 as a crucial downstream effector of HIF‐1α that is both necessary and sufficient for RIPC‐induced repair. Furthermore, we elucidate that NTN4 exerts its dual pro‐angiogenic and pro‐neurogenic functions via integrin β1, activating distinct PI3K/AKT and MAPK/ERK pathways in endothelial and neural cells. This work provides a unified molecular pathway connecting the systemic RIPC stimulus to local cerebral angiogenesis and neurogenesis, offering new mechanistic insights and potential therapeutic targets for post‐stroke recovery.

## Author Contributions

Visualisation: Zhaowei Feng; Writing – original draft: Zhaowei Feng, Kaishen Zhu; Investigation: Zhenqian Liu, Ruiqin Yao, Xiue Wei, Haiyan Liu; Methodology: Zhenqian Liu, Siyu Tang; Formal analysis: Meihua Pan, Chunyu Wang; Data curation: Yiwei Liu; Funding acquisition: Ruiqin Yao; Writing – review and editing: Haiyan Liu.

## Funding

This work was supported by the Jiangsu Commission of Health (M2021027), Xuzhou City health Commission youth project (XWKYHT20230029), the National Natural Science Foundation of China (82471754), The Open Research Project of Key Laboratory of Jiangsu Province Universities (XZSYSKF2023027, XZSYSKF2025017).

## Conflicts of Interest

The authors declare no conflicts of interest.

## Supporting information


**Figure S1:** RIPC augments post‐stroke angiogenesis and neurogenesis. (A) Representative images of CD31 immunofluorescence (red, microvessels) and BrdU/NeuN double immunofluorescence (green/red, newborn neurons). (B) Vascular density was quantified by analysing the CD31^+^ area in the peri‐infarct cortex. (C) The number of newborn neurons was quantified by counting BrdU^+^/NeuN^+^ double‐positive cells. (D) CD31 protein expression was assessed by western blot analysis with densitometry. Bars = 100 μm. *p < 0.05, **p < 0.01, ***p < 0.001 (*n* = 4 biologically independent animals per group).

**FIGURE S2:** RIPC treatment attenuated neuronal degeneration and apoptosis in the cortex and striatum following MCAO. (A, B) Histological analysis using HE and Nissl staining assessed neurodegenerative changes in cortical and striatal neurons of MCAO mice. (C) The anti‐apoptotic effect of RIPC was determined by TUNEL staining in the affected brain regions. Scale bars = 100 μm (*n* = 4 biologically independent animals per group).

## Data Availability

The data that support the findings of this study are available from the corresponding author upon reasonable request.
