# Supplementary figures and images for: HIF‐1α/Netrin‐4 Axis Mediates RIPC‐Induced Angiogenesis and Neurogenesis After Ischemic Stroke
Source: J Cell Mol Med. 2026 Apr 5;30(7):e71121. doi: 10.1111/jcmm.71121 (PMC13052010; doi:10.1111/jcmm.71121)

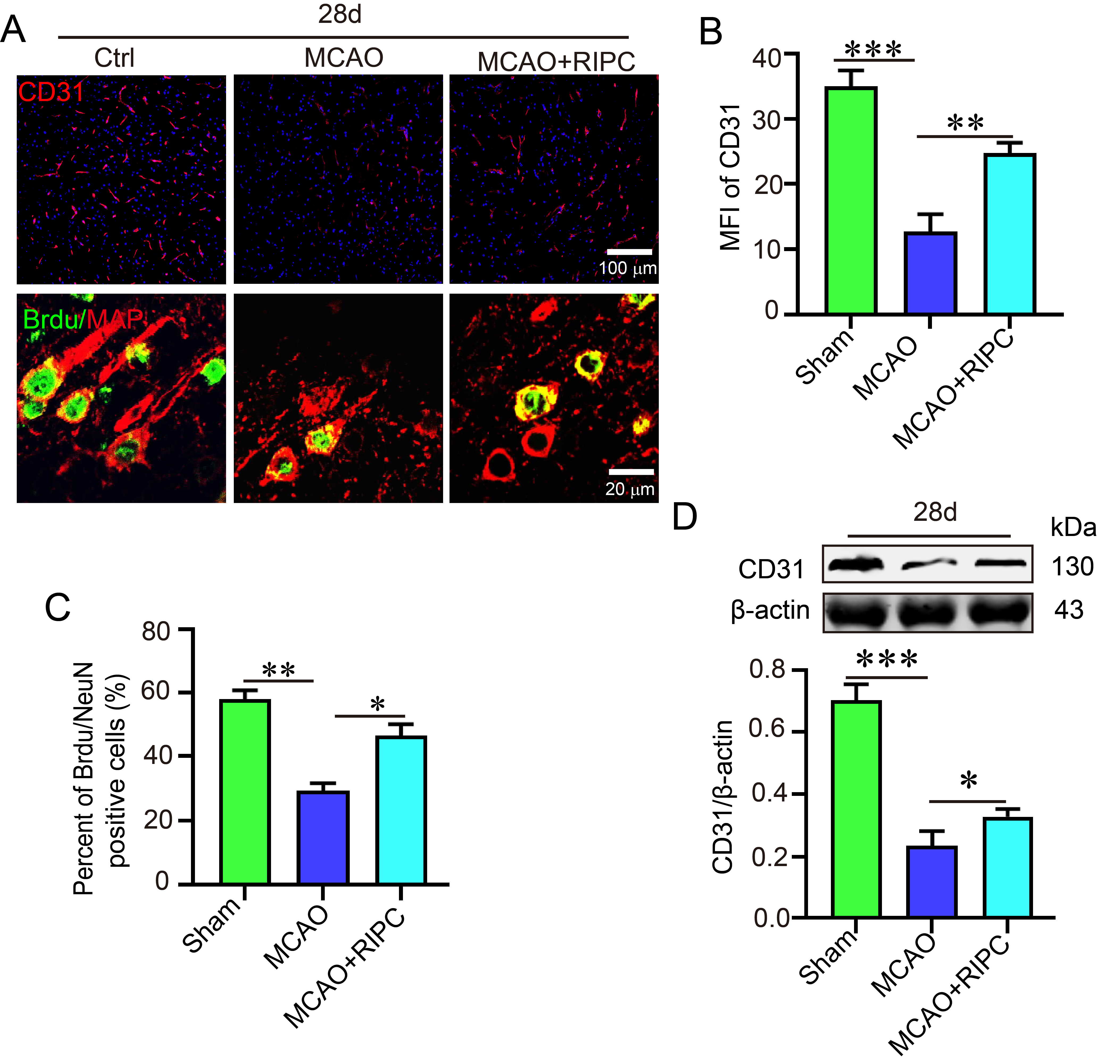

Supplement: Supplementary file 1 — Figure S1: RIPC augments post‐stroke angiogenesis and neurogenesis. (A) Representative images of CD31 immunofluorescence (red, microvessels) and BrdU/NeuN double immunofluorescence (green/red, newborn neurons). (B) Vascular density was quantified by analysing the CD31+ area in the peri‐infarct cortex. (C) The number of newborn neurons was quantified by counting BrdU+/NeuN+ double‐positive cells. (D) CD31 protein expression was assessed by western blot analysis with densitometry. Bars = 100 μm. *p < 0.05, **p < 0.01, ***p < 0.001 (n = 4 biologically independent animals per group). [file JCMM-30-e71121-s002.jpg]

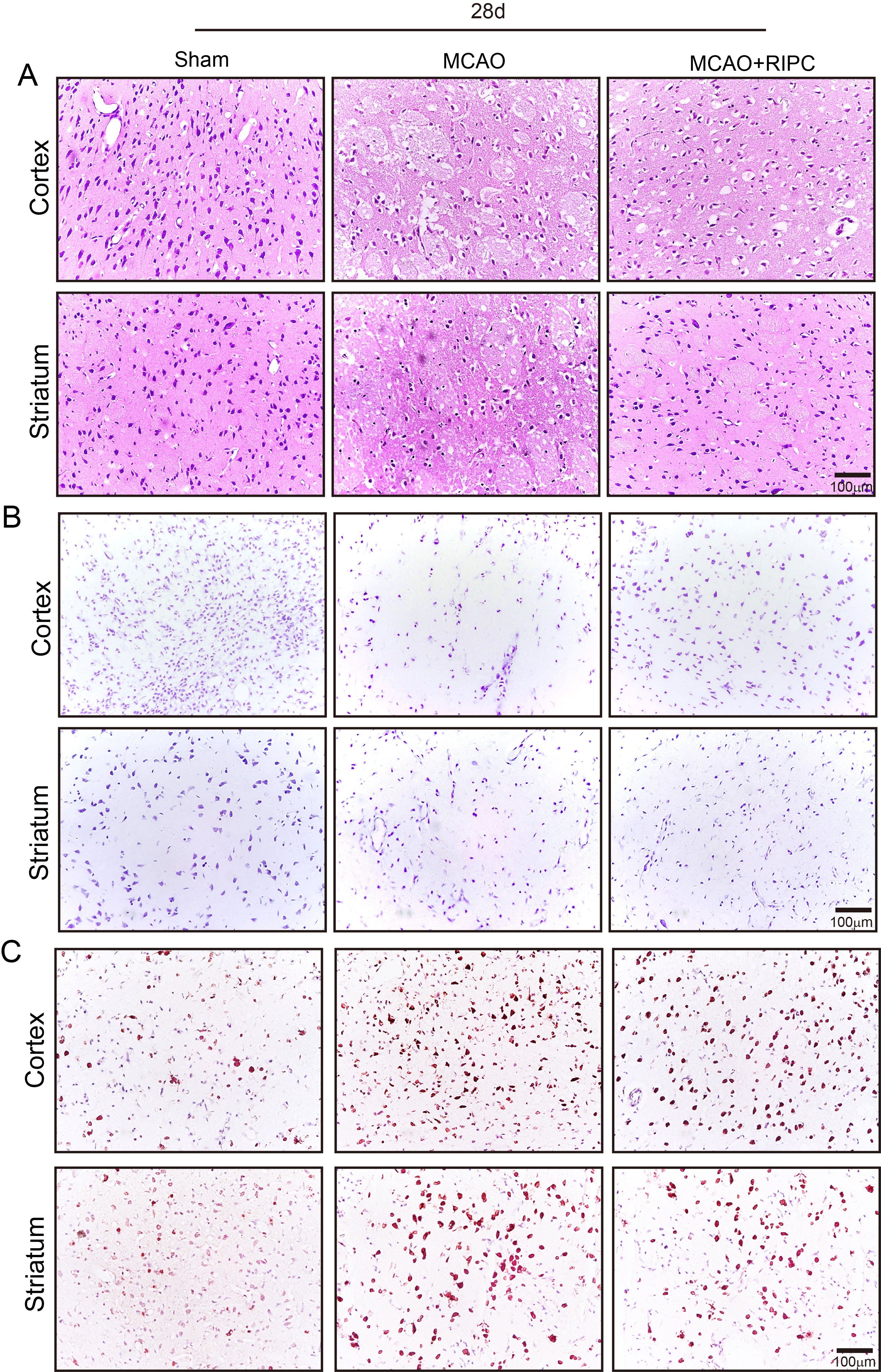

Supplement: Supplementary file 2 — FIGURE S2: RIPC treatment attenuated neuronal degeneration and apoptosis in the cortex and striatum following MCAO. (A, B) Histological analysis using HE and Nissl staining assessed neurodegenerative changes in cortical and striatal neurons of MCAO mice. (C) The anti‐apoptotic effect of RIPC was determined by TUNEL staining in the affected brain regions. Scale bars = 100 μm (n = 4 biologically independent animals per group). [file JCMM-30-e71121-s001.jpg]
